# Studies on the Oxidation Behavior and Microstructural Evolution of Two Nb-Modified HR3C Austenitic Steels under Pure Water Vapor at 650 °C

**DOI:** 10.3390/ma13235447

**Published:** 2020-11-29

**Authors:** Jinlong Wang, Bo Meng, Jintao Lu, Yongli Zhou, Dongxu Yang, Qunchang Wang, Minghui Chen, Fuhui Wang

**Affiliations:** 1 Shenyang National Laboratory for Materials Science, Northeastern University, Shenyang 110819, China; wangjinlong@mail.neu.edu.cn (J.W.); 1910132@stu.neu.edu.cn (B.M.); qcwang@mail.neu.edu.cn (Q.W.); fhwang@mail.neu.edu.cn (F.W.); 2 State Key Laboratory of Coal-Based Clean Energy, Xi’an Thermal Power Research Institute Co., Ltd., Xi’an 710032, China; zhouyongli@tpri.com.cn; 3 Liaoning Non-Ferrous Geological Exploration and Research Institute Co., Ltd., Shenyang 110819, China; lanbeier0216@163.com

**Keywords:** austenitic stainless steel, steam oxidation, oxidation behavior, microstructural evolution

## Abstract

The steam oxidation behavior of three heterogeneous HR3C alloys was investigated at 650 °C comparatively. After a long-term oxidation process for 1000 h, the results demonstrated that the commercial HR3C alloy already exhibited a high oxidation resistance. However, the spallation resistance of the oxide scale was low during the initial oxidation period. The addition of a moderate amount of Nb into the alloy (1#HR3C) increased the oxidation resistance of the alloy. In addition, the improvement of the microstructural stability was substantially influenced by solid solution strengthening and fine grain strengthening. However, the addition of excessive Nb could significantly affect the growth model of the oxide scale and negatively affect the oxidation performance and microstructural evolution of the alloy (2#HR3C).

## 1. Introduction

In power plants, the energy efficiency is proportional to the steam temperature and pressure, which hastens the initiation and development of ultra-supercritical (USC) power plants [1,2]. USC refers to the temperature and pressure of the working fluid (here, water) in the boiler. When the temperature of the fluid exceeds 593 °C or the pressure surpasses 22.1 MPa, it is called USC [3]. A USC power plant has the advantages of low coal consumption and adequate environmental protection. As a result, USC power plants are attracting worldwide attention. 

As the temperature and pressure of the working fluid increase, the service environment for the boiler heating components, which include the water-cooled wall, superheater, reheater, and economizer, becomes more aggressive and harsher [4,5,6,7]. To ensure safe application, these heating components should have superior creep strength, excellent thermal strength, high temperature corrosion, and oxidation resistance [8]. As a type of commercial austenitic stainless steel, HR3C has been widely used as materials for critical components in power plant boilers [9,10] such as superheaters [11] and reheaters [12,13]. HR3C is modified from TP310 via the addition of strong carbide/nitride-forming elements such as Nb and N to increase the creep strength [14,15]. Moreover, the oxidation resistance of HR3C is substantially higher than those of the conventional coarse-grained austenitic stainless steels such as TP304H [16]. This is because the extent of Fe-rich scale formation is minimized and thus a Cr-rich layer predominates. Currently, most studies on HR3C have focused on high temperature creep resistance or corrosion resistance [15,17,18,19,20]. Few studies have investigated the changes on both sides.

With increasing service time, cracking or spalling of the oxide scale that formed on the substrate is inevitable. Young et al. reported [21,22] that the oxide scales would crack when Fe-17Cr steel was oxidized in Ar–5O_2_–20H_2_O for 48 h at 700 °C. Therefore, the structures of the substrate alloys will change, which will cause mechanical property degradation and reduce the service life [23]. The preparation of coatings on the surface and the use of composition modification in alloying elements are both convenient strategies for increasing the stability of the alloy structure. However, investigations have reported severe problems regarding element interdiffusion between the coating and the underlying alloy, which have deleterious effects on the mechanical properties, especially when used in USC power plants for thousands of hours [23]. Thus, modification of the alloying elements in the composition is the preferred approach, and a series of studies have been conducted [24,25,26]. Brady reported [24] that niobium was found to be beneficial for alumina scale formation in newly developed creep-resistant austenitic stainless steels. Nguyen et al. reported [25,26] that cerium and manganese improved the resistance to high temperature oxidation of Fe–20Cr–20Ni in dry CO_2_. Cai et al. [27] reported that an increased amount of N in the solid solution could strengthen the matrix and suppress the coarsening of M_23_C_6_ precipitates. Yuan et al. [28] reported that the addition of Co could increase the rupture strength by lowering the stacking fault energy of the matrix. The addition of a suitable amount of Nb into HR3C austenitic stainless steel can significantly increase the strength of the steel, which reduces the dispersions of Cr_23_C_6_ compounds, and then the solid solution of Nb in the case of the low carbon equivalent of M_23_C_6_ [29]. However, the mechanism and synergistic effect of element modification on the oxidation behavior and mechanical proprieties remain unclear.

In this study, niobium and other elements were modified in commercial HR3C with the primary objective of increasing the mechanical stability and oxidation resistance. However, unexpected effects of niobium on the degradation of the scale and the oxidation behavior were observed. As a result, comparative studies on the oxidation behavior and microstructural evolutions of a commercial HR3C alloy and two types of modified HR3C were conducted at 650 °C under steam oxidation for 1000 h. The effects of niobium on the oxidation behavior and the mechanical properties are discussed.

## 2. Materials and Methods 

In total, three materials were exposed: a commercial HR3C alloy and two modified HR3C alloys. These three types of HR3C steels were designed and produced by the Xi’an Thermal Power Research Institute. The compositions of the HR3C alloys were analyzed using an ICP (inductive coupled plasma) spectrometer and are presented in Table 1. For convenience, the modified HR3C steels were denoted as 1#HR3C and 2#HR3C. These HR3C ingots were prepared by VIM (vacuum induction melting) and homogenized at 1200 °C to achieve an excellent alloy balance, then were hot-forged and rolled at 1170 °C. The specimens for these experiments were cuboids with nominal dimensions of 10 mm × 10 mm × 2 mm. They were ground consecutively with #120, #240, #400, and #600 SiC papers and degreased using an ultrasonic cleaner in acetone and ethanol. 

**Figure 1:**
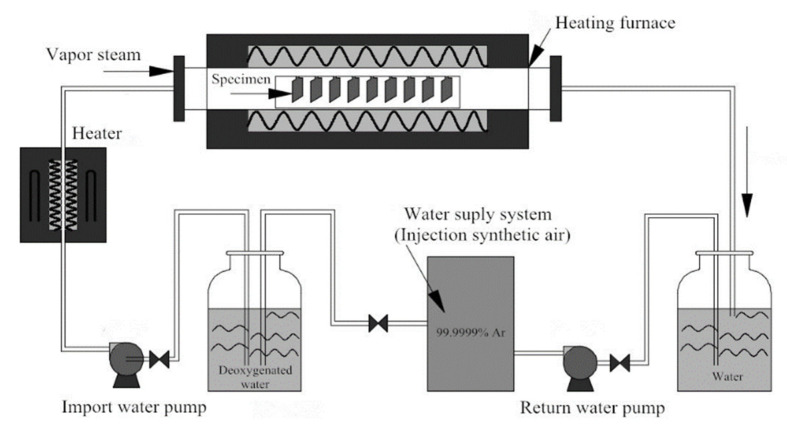
Schematic diagram of the water heating and loop facilities that were used for the steam oxidation experiments.

As shown in Figure 1, the steam oxidation experiment was conducted in a newly built water heat and loop facility for 1000 h. The water heat and loop facilities equipment used for oxidation testing were cooperatively designed by Northeastern University (Shenyang, China) and the Xi’an Thermal Power Research Institute (Xi’an, China). A circulating water system that was coupled with a constant heating source provided a continuous supply of water vapor. The operating parameter of the pressure furnace reached 650 °C and 0.1 MPa. Synthetic air with high purity Ar (99.99% pure) was used as a carrier gas in the water supply system. The inlet conductivity of the water was measured as 0.1 cm/s and the oxygen concentration was less than 6 ppm in the deoxygenated water. Four parallel specimens were all fixed on a Ni–Cr frame and moved into when heated up to 650 °C. After periods of isothermal oxidation, the specimens were taken from the heating furnace. As the specimens were cooled down to room temperature, an electronic balance (0.01 mg precision, Sartorius BP211D) was used to record the mass change of the samples.

The compositions of alloys were confirmed by an inductively coupled plasma emission spectrometer (ICP, ICAP Q, ThermoFisher, Waltham, MA, USA). Phase constituents were characterized by XRD (X’Pert PRO, PANalytical Co., Almelo, Holland, Cu Ka radiation at 40 kV). The obtained x-ray diffraction patterns were recorded in the 2θ range of 10–90°, and a step-scanning mode was employed with a step size of 0.02°. To be noticed, grazing incidence x-ray diffraction (GI-XRD) at incident beam angle of 0.5° was used to analyze the phase constituents of thin scales. The morphologies and microstructures of oxidized samples were characterized by scanning electron microscope (SEM, Inspect F 50, EFI Co., Hillsboro, OR, USA) coupled with an energy dispersive spectrometer (EDS, X-Max, Oxford instruments Co., Oxford, UK) and a transmission electron microscope (TEM, JEM-2100F, JEOL, Tokyo, Japan). Unless otherwise specified, a secondary electron mode was used to examine the surface morphology, and a back-scattered electron mode was used to obtain cross-sectional images of the samples via SEM. During the TEM process, the bright field STEM mode was used to capture the microstructure morphologies and selected area electron diffraction and an energy dispersive spectrometer were used to obtain the phase constituents. Photographs of the metallographic structure after corrosion etching were captured by a digital microscope (B011; Shenzhen Supereyes Technology, Shenzhen, China). The variation in the Vickers hardness with the exposure time was examined using a Micro Hardness apparatus (Blueheler Micrometer5114 Mitutoyo Co., Kanagawa, Japan) with a load of 500 g for 30 s.

## 3. Results

### 3.1. Oxidation Kinetics

**Figure 2:**
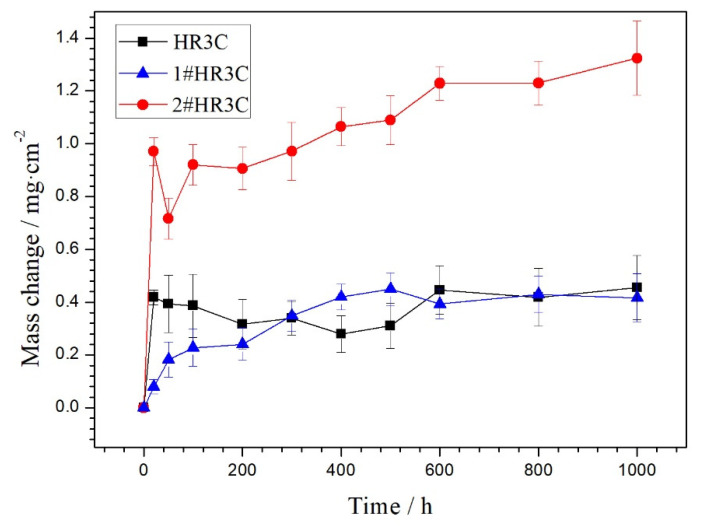
Steam oxidation kinetics of the three groups of HR3C alloys at 650 °C for 1000 h.

Figure 2 shows the oxidation kinetics at 650 °C of the three groups of alloys. All specimens showed a fast initial oxidation stage, followed by a slower steady stage. However, differences were readily observed among the three groups. The commercial HR3C alloy increased the most in weight within the initial 50 h of oxidation. After that period (from 50 to 400 h oxidation), the mass gain slowly decreased as the oxidation test proceeded to a minimum mass gain of 0.316 mg/cm^2^. Multiple spallations occurred on the oxide scale that had formed on the alloy substrate, which resulted in severe fluctuations in mass. Compared with the mass gain of the commercial HR3C alloy, the mass gain of the modified 1#HR3C alloy exhibited a similar but more stable kinetic curve for 1000 h oxidation testing. It showed the most stable mass change trend among the three groups. It strictly obeyed a sub-parabolic law within the first 500 h and reached a maximum mass gain of 0.449 mg/cm^2^. After that, a minor decrease in weight was observed. It is inferred that slight spallation subsequently occurred. The 2#HR3C alloy exhibited a sharp mass decrease in the initial 50 h and showed the highest growth rate of oxides, while unstable oxidation occurred on it according to an overview of its oxidation kinetic results. After the whole oxidation test for 1000 h, the mass gain of the commercial HR3C and 1#HR3C alloys was only one-third of the 2#HR3C mass gain.

### 3.2. Phase Constituents

**Figure 3:**
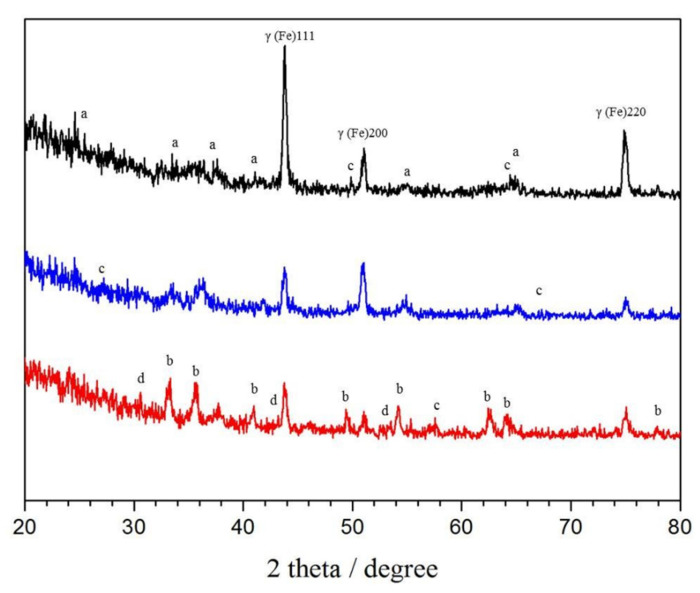
X-ray diffraction (XRD) patterns of the three groups of HR3C alloys after exposure to steam oxidation at 650 °C for 1000 h (a: Cr_2_O_3_, b: Fe_2_O_3_, c: Nb_2_O_5_, and d: FeCr_2_O_4_).

GI-XRD patterns of the commercial HR3C and modified 1#HR3C and 2#HR3C alloys after oxidation at 650 °C for 1000 h are shown in Figure 3. Since the oxide scale was extremely thin, the strongest peaks still corresponded to the metallic phase (austenite γ phase). The oxide scales that formed on the commercial HR3C and 1#HR3C alloys were almost completely composed of the same oxides, except that moderate amounts of Nb oxides were observed within the scale. The oxide scale was composed mainly of Cr_2_O_3_ for the HR3C and 1#HR3C alloys and mainly of Fe_2_O_3_ for the 2#HR3C alloy. The Nb_2_O_5_ peaks were stronger for 2#HR3C than for the others.

### 3.3. Microstructural Analysis

**Figure 4:**
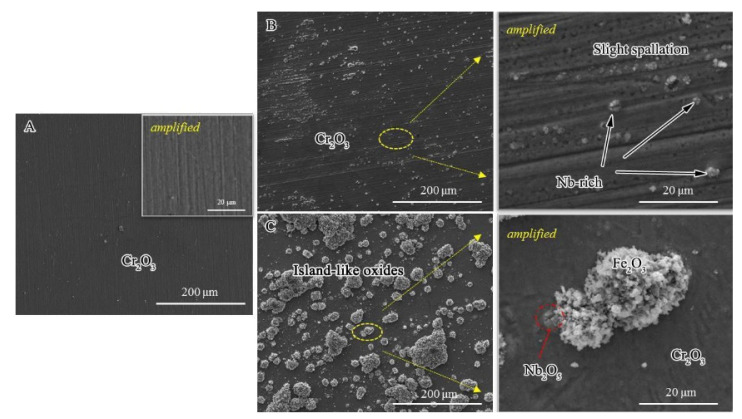
Surface morphologies of the alloys after oxidation in pure water vapor at 650 °C for 1000 h: (**A**) commercial HR3C alloy, (**B**) 1#HR3C alloy, and (**C**) 2#HR3C alloy.

Figure 4 shows the surface microstructure morphologies of the HR3C, 1#HR3C, and 2#HR3C alloys after oxidation at 650 °C for 1000 h. As shown in Figure 4A, the oxide scale that formed on the HR3C alloy surface was flat and uniform. Many metal grooves remained after polishing and grinding, and they remained visible after 1000 h of oxidation; hence, the scale that formed on the surface of the alloy was extremely thin. Compared with the HR3C alloy, the modified 1#HR3C alloy showed a similar surface morphology after oxidation at 650 °C for 1000 h, as shown in Figure 4B. Combined with the XRD analysis and EDS results, a thin Cr_2_O_3_ scale was forming on the surface, while spallation pores were observed on the 1#HR3C alloy. In addition, precipitates appeared on the surface of the 1#HR3C alloy. An amplified image of these precipitates that was obtained via EDS showed that they were mainly composed of Nb-rich phases and that cracks were generated within the niobium oxides. Compared to the commercial HR3C and 1#HR3C alloys, the modified 2#HR3C alloy exhibited different surface morphologies and phase components. The oxide scale was mostly flat with Cr_2_O_3_- except for globular nodules of island-like oxides that had grown on the surface of the alloy. With the help of EDS analysis, these brighter convex oxides were determined to be Fe_2_O_3_. Relatively small and dark oxides of Nb were also observed in the amplified surface image. After comparison, it was inferred that the oxide scale was thicker than those that formed on the other groups, where no distinct grooves were observed. This was in accordance with the oxidation kinetics.

**Figure 5:**
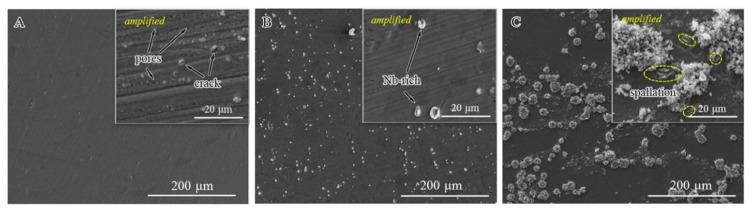
Surface morphologies of the alloys after oxidation in pure water vapor for the initial 50 h: (**A**) commercial HR3C alloy, (**B**) 1#HR3C alloy, and (**C**) 2#HR3C alloy.

To investigate the transformation process of the HR3C alloys in detail, the surface morphologies after exposure to water vapor at 650 °C for 50 h are shown in Figure 5. These showed similar morphological characteristics to the long-term oxidation results. Nevertheless, pores and slight spallation were observed on the surface of the commercial HR3C alloy after 50 h (amplified in Figure 5A), which showed a surface morphology that differed from that of the sample that had been exposed for longer. This might be due to the thickening of the oxide scale, as the locations of these defects became difficult to observe. Furthermore, bright niobium oxides were observed on the 1#HR3C alloy in Figure 5B. For the 2#HR3C alloy, the short-term results were similar to the long-term surface morphology results, except that the island-like oxides were fewer and much smaller. As shown in Figure 5C, the EDS results demonstrated that the oxides in the bulgy area were enriched with Fe, while those in the flat area were enriched with Cr and a moderate amount of Nb. Based on the amplified image, many cracks and spallation areas were identified on the scales, especially at the location where niobium oxides formed.

**Figure 6:**
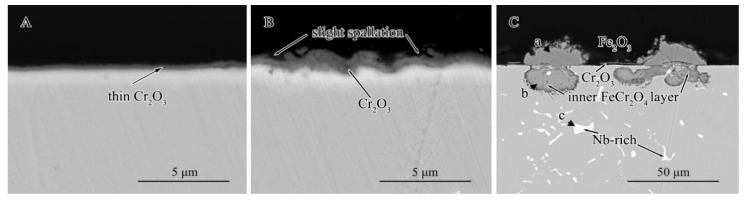
Cross-sectional morphologies of the three groups of alloys after oxidation in pure water vapor at 650 °C for 1000 h: (**A**) commercial HR3C alloy, (**B**) 1#HR3C alloy, and (**C**) 2#HR3C alloy.

The cross-sectional microstructures of the commercial HR3C, 1#HR3C, and 2#HR3C alloys after oxidation at 650 °C for 1000 h are shown in Figure 6. Corresponding to the surface microstructure, the cross-sectional morphologies of the HR3C and 1#HR3C alloys were relatively similar, and a thin layer of Cr_2_O_3_ scale was formed on the alloys. Slight spallation had occurred on 1#HR3C after 1000 h. For the 2#HR3C alloy, the oxide scale had been divided into two layers: The outer grown layer was composed of a thin Cr_2_O_3_ layer and a convex Fe_2_O_3_ layer. The inner layer was rich in Cr and Fe (point b shown in Table 2), it might refer that serious internal oxidation of iron and chromia had occurred and FeCr_2_O_4_ spinel formed. Furthermore, cracking was observed between the two layers.

**Figure 7:**
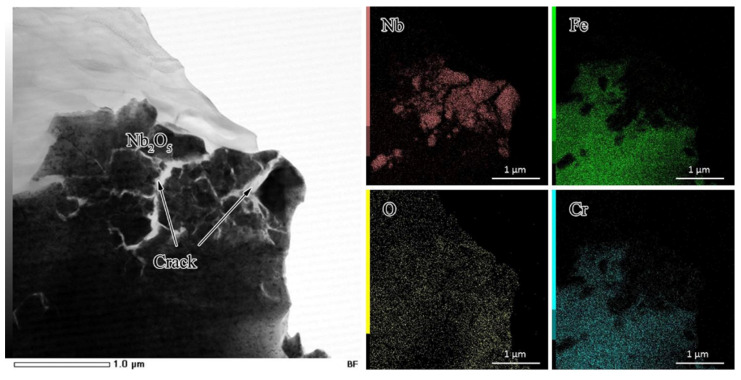
Transmission electron microscope (TEM) microstructures and elemental mappings of the oxide scale on the 2#HR3C alloy after oxidation in pure water vapor at 650 °C for 1000 h.

To investigate the microstructure of the oxide scale on the 2#HR3C alloy after oxidation for 1000 h in detail, the TEM bright field image and EDS mapping results are shown in Figure 7. Judging from the integrity of the surface transmission sample adhesive and the dark oxides that participated on the surface morphologies, as shown in Figure 4, the sampling positions of the thin area after ion thinning process were concentrated on the surface of the sample. The formed oxides were brittle and breakages were observed between the two layers of oxide. Combined with the XRD and EDS mapping results, the oxides were divided into two regions: the shattered region of the oxides was rich in niobium, which reveals that Nb_2_O_5_ was especially enriched there. At other places outside the shattered region, no concentration of Nb was observed and these regions were composed mainly of Fe and Cr oxides.

### 3.4. Microstructural Evolution

**Figure 8:**
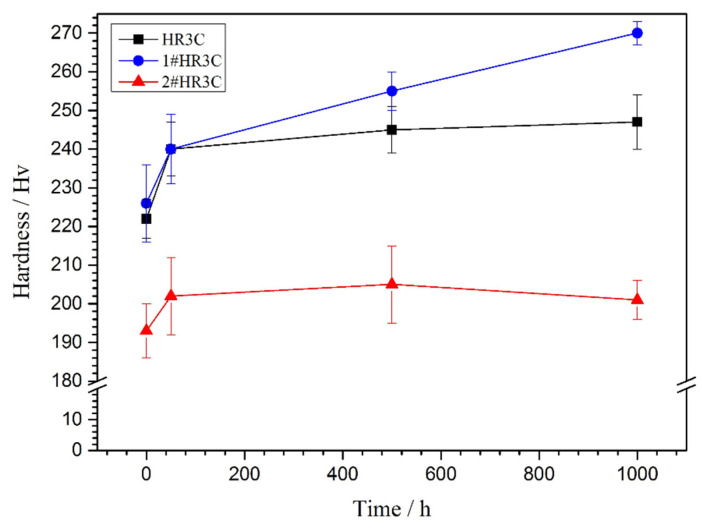
Variations of the microhardness of the three groups of HR3C alloys with the exposure time in a high-temperature water vapor environment at 650 °C.

To investigate the effects of long-term oxidation on the mechanical properties of the alloys, the variations of the Vickers hardness of the three groups of alloys with the exposure time were examined, as shown in Figure 8. It is hypothesized that the mechanical properties of the alloys will be substantially affected by the high temperature oxidation, which is mainly due to the imbalance in the original substrate phases that are transformed into newly generated phases, which is caused by the consumption of scale-forming elements. The hardness of the modified 2#HR3C alloy before the oxidation test was 13.98% lower than that of the commercial HR3C alloy when more than five times the amount of Nb element was added. In contrast, the microhardness of the 1#HR3C alloy with a small amount of Nb was the highest among the three groups, namely, 227 Hv. The microhardness of the three alloys increased rapidly from 0~50 h. However, the three groups of HR3C alloys showed different trends after that period. With the oxidation time increasing, the microhardness of the commercial HR3C alloy remained almost unchanged, while that of the 1#HR3C alloy increased gradually. For the 2#HR3C alloy, the microhardness initially increased and subsequently decreased with prolonged exposure time. After oxidation for 1000 h, the hardness of the 1#HR3C alloy increased from 227 to 270 Hv, which represents an increase of approximately 20%, while the hardness of the 2#HR3C alloy increased from 193 to 200 Hv, which represents an increase of only approximately 3.5%.

**Figure 9:**
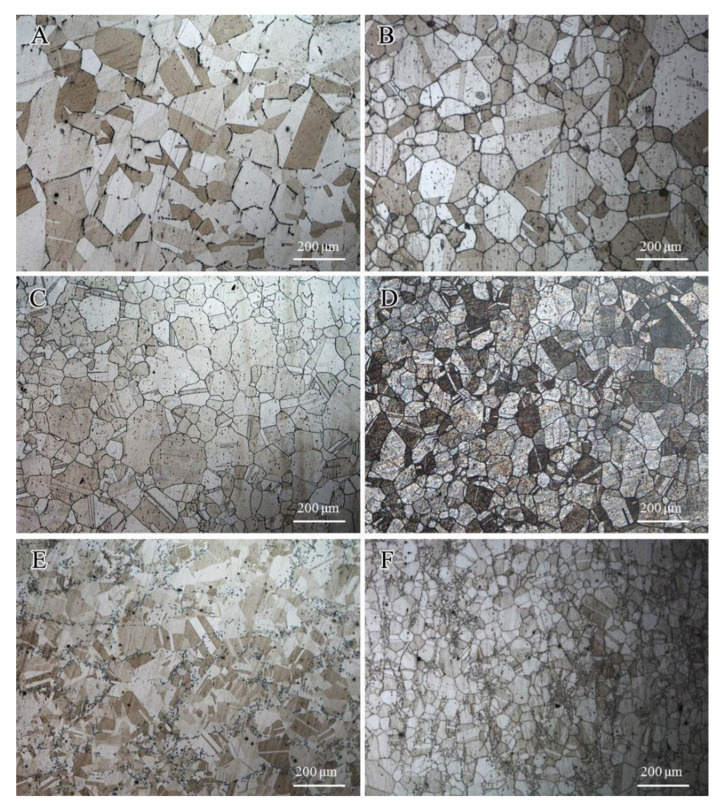
Optical micrographs of the three groups of HR3C alloys before and after steam oxidation at 650 °C for 0 h and 500 h: (**A**,**B**) commercial HR3C alloy, (**C**,**D**) 1#HR3C alloy, and (**E**,**F**) 2#HR3C alloy.

Figure 9 shows corrosion etching images of the metallographic structure on the HR3C and modified HR3C alloys, which explain the changes in the average grain size of the three groups of alloys before and after oxidation at 650 °C for 500 h. Prior to capturing the metallographic images, the oxide scales that formed on the surfaces of the specimens were slightly rubbed off. As Nb and Co solid solutions were added into the alloys, the overall grain sizes of the specimens had become much smaller than that of the commercial alloy. Hence, the original grain size of the HR3C alloy was the maximum, and that of the 2#HR3C alloy was the minimum before the test. With the prolongation of the oxidation time, the grain size decreased, especially for the modified HR3C alloys. Compared with the commercial HR3C alloy, the grain size of the 1#HR3C and 2#HR3C alloys had decreased by almost 1.2 and 2.6 times, respectively. After long-term oxidation, the matrix structures of the modified HR3C alloys changed significantly. Many recrystallizations and second phases precipitated out, while the phase boundaries became smaller and the sizes became uniform.

**Figure 10:**
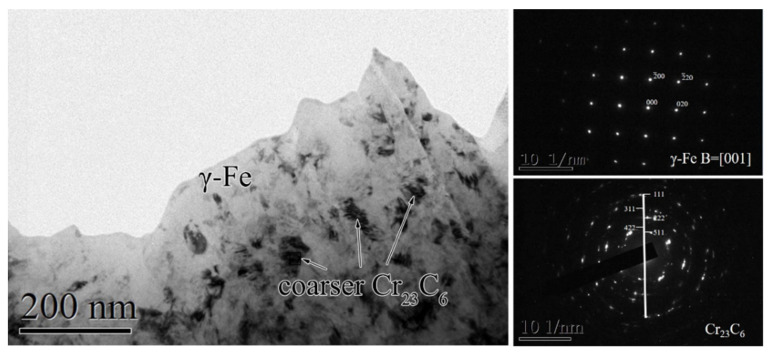
TEM microstructure of the 2#HR3C alloy and its SAED patterns for γ-Fe and coarser Cr_23_C_6_ phases after exposure to pure water vapor at 650 °C for 1000 h.

In order to explore the mechanical properties of the 2#HR3C alloy and their significant fluctuations in detail, the transmission high-resolution results of its microstructures after 1000 h were examined, as shown in Figure 10. Combined with the SAED (selected area electron diffraction) results, the γ-Fe was the main constituent phase. Furthermore, dark spot-like phases were observed in the substrate that corresponded to the Cr_23_C_6_, and the formation of these coarser phases are discussed in the next section.

## 4. Discussion

### 4.1. Steam Oxidation Behavior of the HR3C Alloys

Numerous studies have reported that the water vapor would accelerate the oxidation process of ferrite and austenite Fe-based alloys [30,31]. When oxidized in air at high temperature, the concentration of chromium (25 wt.%) in HR3C steel is enough to sustain the formation of a protective scale [32,33]. In our previous study [34], there were at least three possible ways to cause the cracking of the chromia scales in a water vapor atmosphere. In short, the stress generated in the oxide scales and the decreased deformation of chromia during the oxidation process [16,35].

In our current study, the commercial HR3C alloy had suffered a certain degree of oxidation after a short exposure for 50–500 h at 650 °C. As shown in Figure 2 and Figure 4, the mass change exhibited a slow decline, and slight spalling areas appeared on the surface of the commercial HR3C alloy. The failure mode and mechanism of Cr such as evaporation or spallation have been widely studied and reported [9,36]. It can be speculated that the rapid decomposition of the initially formed protective chromium oxide scale is due to the formation of volatile chromium-containing species within 50 h. The volatilization of Cr occurs in the form of CrO_3_(OH)_2_. The reaction not only requires the presence of water and oxygen in the reaction atmosphere, but temperature is also one of the most important factors [37]. In addition, as the minimum value of P_H2O_·P_O2_ reached 3/4 in this reaction furnace, the evaporation of Cr-species can be ignored. Therefore, the main reason for the decrease in mass was spallation of the oxide scale within 50 to 350 h, which might be related to the stress concentration in different directions during the thickening process of the oxide scales. When the oxidation process was prolonged to 1000 h, minor spallation did not affect the oxidation resistance of the HR3C alloy at 650 °C. A relatively dense layer of Cr_2_O_3_ scale grew on the surface of the substrate alloy, which was significantly related to the reason for the slow growth of the commercial HR3C alloy after 300 h.

Compared with the commercial HR3C alloy, the modified 1#HR3C alloy exhibited more stable oxidation resistance during the first 500 h, and both the oxidation kinetics and the mass gain were the lowest within the three groups. Several studies showed that there were two strategies for improving the formation of high-quality oxide scale: (1) modifying the elemental components of the alloy to change the diffusion mode of Cr/O, and (2) grain refinement to reduce the critical Cr concentration for scale formation [38,39]. Mitsutoshi et al. [40] indicated that the critical Cr concentration in a wet atmosphere increased approximately 1.2 times higher than that in a dry environment. Compared with the commercial HR3C alloy, although the content of Cr in the 1#H3RC alloy did not differ substantially, the modification of approximately 5% by weight of Nb, Co, and other elements changed its organizational structure. This will be discussed in detail in the next part. The other process is a substantial reduction in the grain size of the matrix, which utilizes selective oxidation of the alloy. Grain boundaries are important paths for short-range diffusion. For alloys with a high Cr content such as HR3C, grain refinement can significantly improve the selective oxidation of Cr on the alloy surface, and rapidly form a protective oxidation at high temperature. Figure 7 shows that the grain sizes of the modified HR3C alloys were much smaller than that of the commercial alloy, which was only approximately 200 μm. Otsuka et al. [41] quantitatively showed that the critical Cr concentration to form a dense scale had dropped approximately 20 wt.% on an austenitic stainless steel with grains ranging from 110 to 20 μm in a wet atmosphere. It can be speculated that the grain size reduction and minor element addition play an important role during the oxidation process. In addition, the 1#HR3C alloy showed thin and continuous microstructure morphologies in Figure 4 and Figure 5. The fine grain structure provided the diffusion channels and oxide nucleation sites for Cr; thus, this alloy exhibited the best oxidation resistance among the three groups during the first 500 h. However, minor trends of mass decrease and pores were observed in the kinetics and surface morphologies in the final process, which might due to the strong influence of long-term oxidation in the vapor environment for 1000 h. After that, a Cr depletion region was formed beneath the oxide layer. Unexpected cracking and buckling finally exhibited as the substrates were insufficient for healing and sustaining the intact protective scale.

For the 2#HR3C alloy, pores and nodule oxides were distinctly observed on the surface after oxidation. The number and size of the nodule oxides increased with the prolongation of the oxidation experiment, which corresponded well with the data of oxidation mass change in Figure 2. After longer exposure, the nodule oxides were expected to spread across the surface and form double-layered scales. These consisted mainly of less protective spinel and oxides of iron and niobium. In addition, many cracking and spallation areas were identified. The cracking and spallation of the protective chromium oxide scale led to weak interfacial adhesion. Thus, cracking was observed at the interface of the two oxide layers, and an internal oxidation process occurred. The cracks changed the oxidation process behavior of the HR3C from external to internal. As observed on the cross-sectional morphology in Figure 6, the spinel of Cr_2_O_3_ and iron precipitated in the metal through the inward diffusion of oxygen and the outward. Internal oxidation prevail was observed as the diffusion of Cr was predominantly affected by the oxygen permeability and the diffusion coefficient of chromium in the alloy. Compared with the commercial HR3C and 1#HR3C alloys, the excessive content of Nb and its oxide precipitation in the modified 2#HR3C alloy might be the main reason for the instability of early rapid oxidation and the transformation of the oxidation formation to an internal process. In addition, the development of spinel would be formed and controlled by oxygen diffusion along the grain boundaries.

### 4.2. Effects of Nb Modification on the Steam Oxidation Behavior

As a refractory element, niobium is often added to the elemental compositions of alloys for solid solution strengthening. The effects of Nb on the mechanical behaviors of alloys have been reported by many researchers [42,43]. Pint and Guo et al. [26,43,44,45] demonstrated that the addition of elements with large atomic radius such as Y, Zr, Ce, La, and Hf could significantly affect the oxidation behaviors of alumina-forming alloys, thus, improving the scale adhesion and oxidation performance. In our recent studies [46,47], the refractory element of Ta could also change the segregation state between grain boundaries, thereby resulting in a significant change in the formation mechanism of oxide scale, namely, a change in the diffusion mode of oxide scale from internal oxidation to external oxidation, similar to the reactive element. Nb oxides affect the stability of protective coatings such as chromium oxides. 

In this study, the contents of Nb in the modified HR3C alloys were 0.59 and 2.36 by weight, which were approximately 0.37 and 4.81 times larger, respectively, than that of the commercial alloy. In addition to changing the main compositions of the oxides, it was found that the addition of Nb generated niobium oxides in the oxide scale. According to the value of the Gibbs free energy and its Ellingham–Richardson diagram under 650 °C, niobium can form its oxide Nb_2_O_5_ in this process. Moreover, this was confirmed by the SEM/EDS and TEM results in Figure 4 and Figure 5. Although the amount and area of Nb_2_O_5_ were not substantial compared with the main constituents of the oxides, it was found on the surface of the modified alloys immediately after 50 h. The outward diffusion of Nb was through the grain boundaries in the alloy or oxides. As the atomic radius of Nb was large, the diffusion ability might be limited under 650 °C in the test. Therefore, there was no significant difference in the amount of Nb oxide between 50 h and 1000 h. The effect of oxides that were formed by refractory elements on the oxidation behavior has frequently been considered in other studies such as Ta oxides [46,47], W oxides [48], and Y oxides [49]. For example, the creep lifetime could be significantly increased by co-adding Nb, Ti, and V together. However, this always had a negative effect on the oxidation behavior. The oxides of refractory elements were mostly unstable and prone to spalling or evaporation in a harsh high temperature environment. As shown in Figure 8 and Figure 9, the amplified morphologies of the Nb oxides were fragmented and cracked. The oxides were less protective. This was because the oxides of Nb were brittle, and their lattice constant (crystal structure) and thermal expansion coefficients did not match those of Fe_2_O_3_ or Cr_2_O_3_, and they were likely to break up and crack at the scale-forming interface. Shen et al. [50] also reported that the breakaway of oxide layer might be caused by micro-cracks and channels. The voids or defects that were left by the cracking or peeling of Nb_2_O_5_ resulted in the subsequent precipitation and production of non-protective Fe oxides, which provided diffusion paths for the internal diffusion of O. The oxidation performance depends on the diffusion process of Cr and O. Usually, the diffusion rate of Cr^3+^ is prone to be being affected by the vacancy concentration and temperature. The addition of Nb in HR3C alloys might have a significant influence on the vacancy formation energy of Cr, thereby affecting the external oxidation behavior of Cr.

### 4.3. Effects of Element Modification on the Mechanical Properties

The original objective of adding Nb was to improve the mechanical properties of the HR3C alloy. The creep-resistance properties of HR3C alloys were prone to being affected by the precipitation behaviors during the isothermal aging and oxidation process. The precipitation hardening of nano-sized MC-type (with a cF-NaCl structure, NbC) carbides substantially increased the creep resistance. In the optical micrographs of the three groups of alloys in Figure 9, several phase boundaries and dislocation structures were observed in the modified 1# and 2#HR3C alloys. It was speculated that the addition of Nb led to the substantial decrease in the grain size of the alloy. In addition to MC carbides, excessive Nb might lead to the formation of coarse Fe_2_M-Laves phases, while less Nb leads to the formation of Cr_23_C_6_ [43]. The moderate amount of Nb in the 1#HR3C alloy led to the highest microhardness among the three groups due to a large amount of second-phase precipitation. However, as shown in Figure 10, phases that dark void-like microstructures were observed in the 2#HR3C alloy, which was identified as the primary NbC particles. Coarse harmful brittle phases such as Cr_23_C_6_ and σ-FeCr particles were always precipitated from the austenitic alloy after long-term aging at 600–900 °C. Since these brittle phases act as a path for crack propagation at the grain boundaries, especially in a long-term SCW environment at high temperatures, the chain-like distribution of these brittle phases in the chain-like distribution causes a significant decrease in ductility and toughness. Intergranular corrosion was more inclined to happen as the Cr-depletion area extends to a critical size in the substrate alloy [51,52]. In addition, any other harmful mechanical phases such as the G phase (Ni_16_Si_7_M, M = Nb/Ti/Zr, Cf-Mn_23_Th_6_ type) and Laves phase (Fe_2_M, M = Mo/Nb, hp-MgZn_2_ type) [53,54] would be precipitated and have a negative effect on the mechanical properties of HR3C with a excessive modification of Nb.

**Figure 11:**
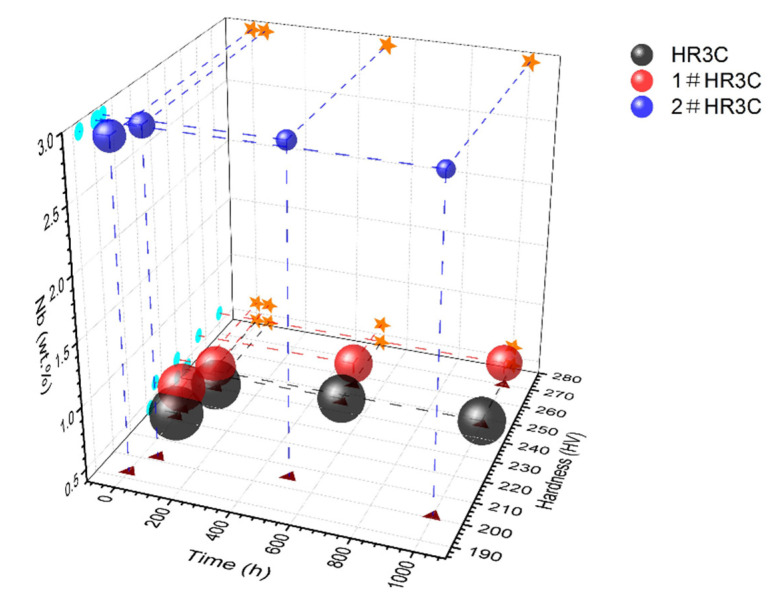
Variations of the average grain sizes of the three groups of HR3C alloys after steam oxidation with the exposure time, where the extent radius represents the grain size in the alloys.

Figure 11 shows the relationship between the average grain size and hardness versus the oxidation time for the alloys. The grain size of the alloy was finer after oxidation, and the hardness changed substantially with the prolongation of the oxidation time. The hardness of the three alloys increased after 50 h, which was mainly due to the change of Cr_23_C_6_ that was caused by oxidation and the precipitation of MC on the grain boundaries. Wen reported that Nb could preferentially interact with C to form NbC to partially replace Cr_23_C_6_. This trend was supported by the thermal calculation [43]. Lu et al. [35] argued that the Nb/C ratio could affect the phase precipitation, however, the hardness changed substantially with the prolongation of the oxidation time. The hardness of the commercial and 1#HR3C alloys remained almost unchanged after 500 h of exposure, whereas that of the 2#HR3C alloys decreased. This was due mainly to the precipitation and exfoliation of the Nb and matrix phases, thereby resulting in significant degradation of the mechanical properties of the alloy. Thus, the modified amount of Nb significantly influenced the phase precipitation process in the HR3C alloy, in which a suitable Nb content would inhibit the precipitation of Fe_2_Nb and Cr_23_C_6_ effectively.

A moderate amount of Co was also added to the modified 1# and 2#HR3C alloys. It has been reported that Co substantially influences the stacking fault energy (SFE) in Ni–Co binary alloys, and therefore also affects the ability to form twins. It plays an important role in increasing the rupture strength by reducing SFE. Furthermore, the low SFE can increase the dislocation spreading width and the dislocation density and promote dislocation entanglement. This results in the increase in the driving force of recrystallization and, thus, increased likelihood of recrystallization. It was also found that the addition of C and Co could play a beneficial role in the formation of the M_23_C_6_ phase, while the effects of Mn, N, and Si on the mechanical proprieties remained unclear. The effects of these elements must be further explored systematically in the HR3C alloy.

## 5. Conclusions

Based on the commercial HR3C alloy, two modified alloys, namely, 1#HR3C and 2#HR3C, were prepared by adding alloying element Co while enhancing the content of Nb. The oxidation behaviors in steam and the evolutions of the microstructure and hardness at 650 °C for 1000 h of the three alloys were investigated comparatively. 

From the above study, the following conclusions can be drawn:

(1) The commercial HR3C alloy exhibited a certain extent oxidation resistance throughout the test. Slight spallation and the formation of pores were observed during the initial oxidation process. Its microstructure and hardness showed high stability under oxidation at 650 °C.
(2) For the modified 1#HR3C alloy, after increasing the Nb content by 37% and adding a moderate amount of Co, its oxidation resistance was slightly enhanced compared to that of the commercial alloy. In addition, its microstructure was stable after oxidation for 1000 h at 650 °C and the hardness was even increased. Increasing alloying elements Co and Nb refined the alloy grains. Solid solution strengthening and fine-grain strengthening contributed to the increase in the hardness.
(3) For the modified 2#HR3C alloy, the Nb content increased by 4.81 times compared to that of the commercial alloy. The oxidation rate was the highest among the three alloys and the oxide scale was more likely to spall off due to the formation of abundant but less-protective niobium oxide at the surface. Although its grain size was the finest, its microhardness was the lowest, irrespective of the fine-grain strengthening. After exposure at 650 °C for 500 h, its hardness even began to decrease slightly.

## Figures and Tables

**Table 1:** Compositions of the three groups of HR3C alloys detected by inductive coupled plasma (ICP) (wt.%).

Alloy.	Ni	Cr	Mn	Nb	Si	N	C	B	P	Co	Fe
Nominal HR3C	20.40	24.65	1.07	0.43	0.47	0.23	0.07	---	0.019	---	Bal.
Commercial HR3C	20.08	23.79	1.01	0.41	0.47	0.21	0.05	---	0.020	---	Bal.
1#-HR3C	18.91	23.3	0.03	0.59	0.44	0.16	0.20	0.002	0.027	4.65	Bal.
2#-HR3C	19.43	20.7	0.03	2.93	0.28	0.13	0.13	0.002	0.018	4.74	Bal.

**Table 2:** Chemical composition (wt.%) measured (denoted in and Figure 6C) by energy dispersive spectrometer (EDS).

No.	O	Fe	Cr	Nb	Bal
a	31.51	62.74	1.09		2.20
b	26.80	28.75	39.35		5.1
c	2.15	1.09	0.84	95.32	0.6
